# A comprehension scale for central-line associated bloodstream infection: Results of a preliminary survey and factor analysis

**DOI:** 10.1371/journal.pone.0203431

**Published:** 2018-09-13

**Authors:** Sushant Govindan, Katherine Prenovost, Vineet Chopra, Theodore J. Iwashyna

**Affiliations:** 1 Department of Medicine, University of Michigan Health System, Ann Arbor, MI, United States of America; 2 Center for Clinical Management Research, Ann Arbor VA Healthcare System, Ann Arbor, MI, United States of America; 3 Patient Safety Enhancement Program, Ann Arbor VA Healthcare System, Ann Arbor, MI, United States of America; University Hospital Jena, GERMANY

## Abstract

**Background:**

Central line-associated bloodstream infections (CLABSI) are associated with significant morbidity and mortality. This condition is therefore the focus of quality initiatives, which primarily use audit and feedback to improve performance. However, feedback of quality data inconsistently affects clinician behavior. A hypothesis for this inconsistency is that a lack of comprehension of CLABSI data by decision makers prevents behavior change. In order to rigorously test this hypothesis, a comprehension scale is necessary. Therefore, we sought to develop a scale to assess comprehension of CLABSI quality metric data.

**Methods:**

The initial instrument was constructed via an exploratory approach, including literature review and iterative item development. The developed instrument was administered to a sample of clinicians, and each item was scored dichotomously as correct or incorrect. Psychometric evaluation via exploratory factor analyses (using tetrachoric correlations) and Cronbach’s alpha were used to assess dimensionality and internal consistency.

**Results:**

97 clinicians responded and were included. Factor analyses yielded a scale with one factor containing four items with an eigenvalue of 2.55 and a Cronbach’s alpha of 0.82. The final solution was interpreted as an overall CLABSI “comprehension” scale given its unidimensionality and assessment of each piece of data within the CLABSI feedback report. The cohort had a mean performance on the scale of 49% correct (median = 50%).

**Conclusions:**

We present the first psychometric evaluation of a preliminary scale that assesses clinician comprehension of CLABSI quality metric data. This scale has internal consistency, assesses clinically relevant concepts related to CLABSI comprehension, and is brief, which will assist in response rates. This scale has potential policy relevance as it could aid efforts to make quality metrics more effective in driving practice change.

## Introduction

Central line-associated bloodstream infection (CLABSI) is a condition that has been the focus of quality metric development and public reporting.[1] This is related to its attributable mortality, significant costs, and complexity regarding prevention efforts.[2–4] Quality metrics, while costly and labor-intensive to develop,[5] are intended to motivate practice change via audit and feedback initiatives.[6, 7] However, for both CLABSI data and quality metrics in general, reporting-based interventions are variably efficacious in impacting clinician behavior.[8–14] To date, the mechanisms generating such variability have not been identified.

Comprehension of CLABSI quality metric data may moderate the efficacy of audit and feedback. In the area of risk interpretation and communication, it is well known comprehension is a factor that impacts medical decision-making and impetus to change behavior.[15, 16] Whether clinician comprehension of CLABSI quality metric data influences practice change is a hypothesis that remains untested. In order to evaluate this hypothesis, an internally consistent scale is required for assessing comprehension. However, no such scale on CLABSI data exists in the literature.

Therefore, we sought to develop a CLABSI comprehension scale. We employed survey data from a previous study that piloted a CLABSI comprehension assessment to clinicians.[17] The goal of this new scale is to reliably measure the adequacy and extent with which front-line clinicians comprehend CLABSI feedback data. CLABSI was chosen given its relatively consistent definition, ubiquitous quality reporting, and policy relevance as a national quality indicator.[18]

## Methods

We utilized a previously employed, broad approach to develop a new survey-based scale of an individual’s comprehension of CLABSI data.[19] This included a combination of literature review, survey methodology, and psychometric data analysis. Individual items were constructed in an iterative process with pilot testing. This was followed by a cross-sectional assessment of CLABSI comprehension and subsequent factor analysis. The study was deemed IRB exempt by the University of Michigan: protocol HUM00106696.

### Development & refinement of initial item pool

First, a literature review was conducted on quality metric comprehension and data presentation methods. Infographic methods utilized by government agencies and two tertiary care centers were also evaluated. The review found little research and no scale development regarding CLABSI quality metric comprehension. A CLABSI data presentation array was therefore adapted for the survey from Rajwan et al, who qualitatively validated their format using physician feedback.[20] Second, an iterative process served to assemble a question bank from which the scale would be constructed. Questions were subsequently evaluated based on clarity, conceptual assessment, and difficulty by the authors (SG, VC, TJI). Fifty-four questions were then narrowed to 11 items that made the initial assessment. Correct answers were defined based on literature review and input from methodological and content experts (TJI and VC). Third, eight hospitalists and intensivists formed a cohort for pilot testing of the instrument. These interviews provided feedback upon which additional revisions were made.

### Survey sample

An 11-item CLABSI comprehension instrument was then deployed to clinicians for preliminary scale development, as previously reported.[17] This was a convenience sample of clinicians, recruited via Twitter and employing Surveymonkey as the platform. Two study authors (TJI and VC) who utilize their Twitter accounts for professional purposes distributed solicitation tweets to their followers. A link to the survey was provided in each tweet, and the period of recruitment was 30 days. Given the nature of Twitter recruitment without a clear denominator, a response rate could not be calculated. To ensure respondents were clinicians, they needed to first answer a screening question recognizing that central lines were placed in the subclavian site but not the aorta, iliac, or radial sites. To prevent item order effects,[21] the order of questions was electronically randomized for each respondent.

### Statistical analysis

The present analysis sought to assess dimensionality via exploratory factor analysis and internal consistency of the resultant factors.[22] Overall performance, item-specific performance, and factor-specific performance were calculated using all available data. Items were excluded if the sample did not exhibit an adequate spread of scores on the question,[23] defined as over 90% of the cohort responding either correctly or incorrectly.

Bartlett test of sphericity and the determinant of the correlation matrix were both calculated to ensure adequacy of the data for factor analysis.[24] Due to the dichotomous nature of the data, the primary factor analysis was done on a tetrachoric correlation matrix rather than a Pearson correlation matrix (which is best used for continuous data).[25, 26] The factor analysis employed data from those participants who completed the entire survey. The underlying assumption was that the instrument’s dichotomous scores represent latent, normally distributed constructs (see S1 Table and S1 Fig for primary data and Stata code). A secondary factor analysis based on Pearson correlations was also carried out to see if differences existed with respect to number of factors and item loadings between the two techniques. Item-rest correlations were also checked to identify potential problem items if they had negative values, i.e., were reverse-coded which, for this scale, would not be accurate.

The analysis identified the number of factors based on eigenvalues greater than 1. However, we did not use this criteria alone for final inclusion, as a final solution based only on eigenvalues can be inaccurate and potentially results in too many factors and overfitting.[27] Thus, other methods, including scree plot examination and clinical interpretability, were also used. Subsequent factor analyses extracted the appropriate number of factors for a final solution. Items with factor loadings ≥ 0.45 were kept;[28] if the final solution contained more than one factor, a rotated pattern matrix was interpreted. The factor(s) in the final model were assessed for unidimensionality via Cronbach’s alpha.[29] In the case of dichotomous outcomes where the assumption of normality is violated, calculation of Cronbach’s alpha via Pearson’s correlation can produce distorted results.[30] Thus, the calculation was adjusted for dichotomous outcomes by employing tetrachoric correlations, a methodology similar to polychoric correlations employed for polytomous variables.[30, 31] Acceptability of the scale was defined as Cronbach’s alpha greater than 0.7, following Nunnally and Bernstein’s recommendation.[32] The factor solution was corroborated by evaluating item-rest correlations between items and the scale; those with values less than 0.2 were omitted, with the final set of items compared to the final factor solution for consistency.[32]

Over the course of several meetings, the authors (SG, VC, TJI) subsequently evaluated the factor solution for clinical relevance. Several areas related to the questions were assessed: wording and semantics, answer choice format, patterns of errors made by respondents, cognitive tasks within items (numeracy vs risk-adjustment understanding vs gist), and type of CLABSI data tested. Analyses were conducted using Stata MP 14.0 (College Station, TX).

## Results

A total of 97 respondents answered at least one question; 72 respondents answered all 11 questions. Therefore, a total of 939 unique responses were available for analysis. Sixty-eight (85%) respondents were from the United States. Thirty-nine (48%) were physicians, and thirty-one (39%) were nurses. There was a range of clinical experience in the sample, with forty-four (55%) respondents having 6–20 years’ experience.

### Item and dataset evaluation

Mean performance of the cohort across all answered questions was 61% correct (SD = 21%). Item accuracies ranged from 17%-95% correct (Table 1). Two questions had at least 90% correct responses and were excluded from subsequent analyses given lack of variation.[23] All remaining items had statistically significant correlations of greater than 0.2 with overall performance. Additionally, the determinant of the correlation matrix was greater than zero (hence, not multicollinear), and the Bartlett test of sphericity rejected the null hypothesis that the observed matrix was equal to the identity matrix (p < 0.001).

**Table 1:** Sample performance on survey questions.

Survey Question	Percent Correct	Number of Respondents
*Which is better: a higher or lower SIR?	95%	86
*Which hospital uses the most central lines?	90%	89
Which hospital has the lowest CLABSI rate?	80%	87
If hospital A doubled its central-line use but other practice patterns remained the same, how many actual infections would hospital A expect to have?	79%	80
If hospital G’s number of actual infections doubled, what would its CLABSI rate be?	77%	83
The presence of a gastrostomy (g) tube is a risk factor for CLABSI. If this variable is not accounted for in CLABSI reporting, how would this impact the interpretation of the number of infections projected by national experience?	75%	87
Which hospital is most effective at preventing CLABSI?	51%	87
If hospital B had its number of projected infections halved, what is its SIR?	46%	80
Suppose hospitals A and H have the exact same CLABSI prevention practices. Which hospital will have the higher number of CLABSI?	34%	87
Which hospital’s patients are the most predisposed to developing CLABSI?	32%	87
Suppose hospital A begins using a central line with an antibiotic coating that halves infections. What would hospital A's number of projected infections be?	17%	78

*Questions with a variation in performance of 10% or less were excluded.

### Factor analysis

Employing principal factor analysis on the tetrachoric correlation matrix, the final solution, or scale, included one factor with an eigenvalue of 2.55. Factor 1 contained four survey questions, or items, that had loadings >0.45 on the factor. The other five questions all had loadings under 0.45 (Table 2) and were thus not included. Factor 1 explained over 40% of the total variance in the reduced question set, and it had a Cronbach’s alpha of 0.82. With respect to cohort performance on the final scale, the mean percent correct on Factor 1 was 49% (median 50%).

**Table 2:** Factor analysis results: Loadings of the pattern matrix for factor solution.

Survey Question	Factor 1	Uniqueness	Item-Rest Correlation
Q6: Suppose hospital A begins using a central line with an antibiotic coating that halves infections. What would hospital A's number of projected infections be?	0.86	0.26	0.35
Q7: Which hospital is most effective at preventing CLABSI?	0.79	0.37	0.53
Q8: The presence of a gastrostomy (g) tube is a risk factor for CLABSI. If this variable is not accounted for in CLABSI reporting, how would this impact the interpretation of the number of infections projected by national experience?	0.70	0.52	0.46
Q2: If hospital G’s number of actual infections doubled, what would its CLABSI rate be?	0.64	0.58	0.36
Q3: Which hospital has the lowest CLABSI rate?	0.10	0.99	
Q4: If hospital B had its number of projected infections halved, what is its SIR?	0.24	0.94	
Q5: If hospital A doubled its central-line use but other practice patterns remained the same, how many actual infections would hospital A expect to have?	0.33	0.89	
Q10: Which hospital’s patients are the most predisposed to developing CLABSI?	0.18	0.97	
Q11: Suppose hospitals A and H have the exact same CLABSI prevention practices. Which hospital will have the higher number of CLABSI?	0.28	0.92	

**Fig 1:**
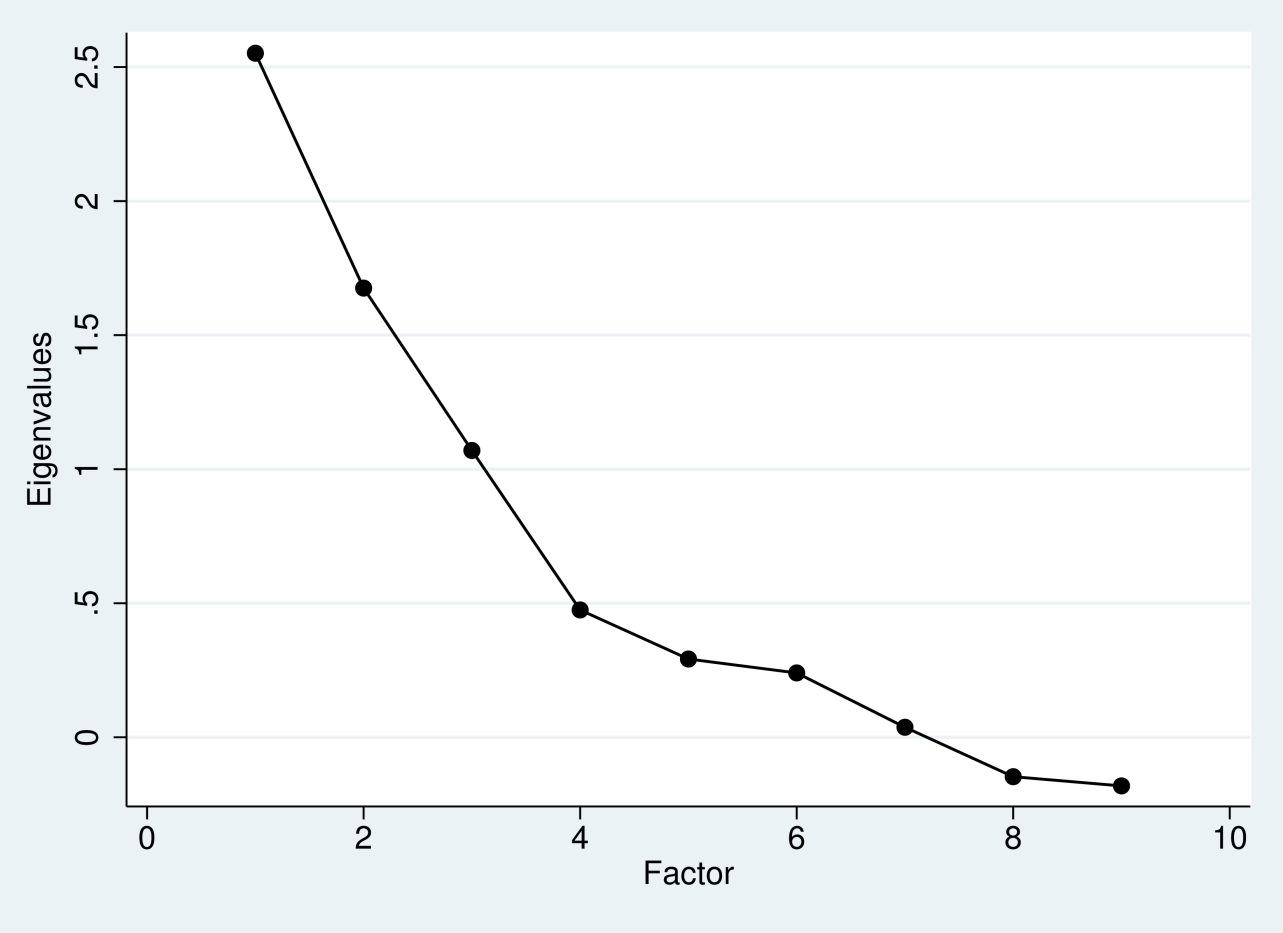
Scree plot: Eigenvalues as a function of number of factors extracted.

Two additional factors had eigenvalues greater than 1: 1.67 and 1.07, respectively. However, there were several additional findings that favored a one-factor solution. First, the scree plot **(**Fig 1**)** suggested a large difference between Factor 1 and the other factors. Second, Factor 1 succinctly assessed all concepts that the authors judged clinically pertinent, and did so with internal consistency. Third, item-rest correlation analysis corroborated the one-factor solution, with all items having similar values that were greater than 0.3 (Table 2). Finally, a secondary factor analysis based on Pearson’s correlations agreed with a single factor solution with the same item loadings.

**Fig 2:**
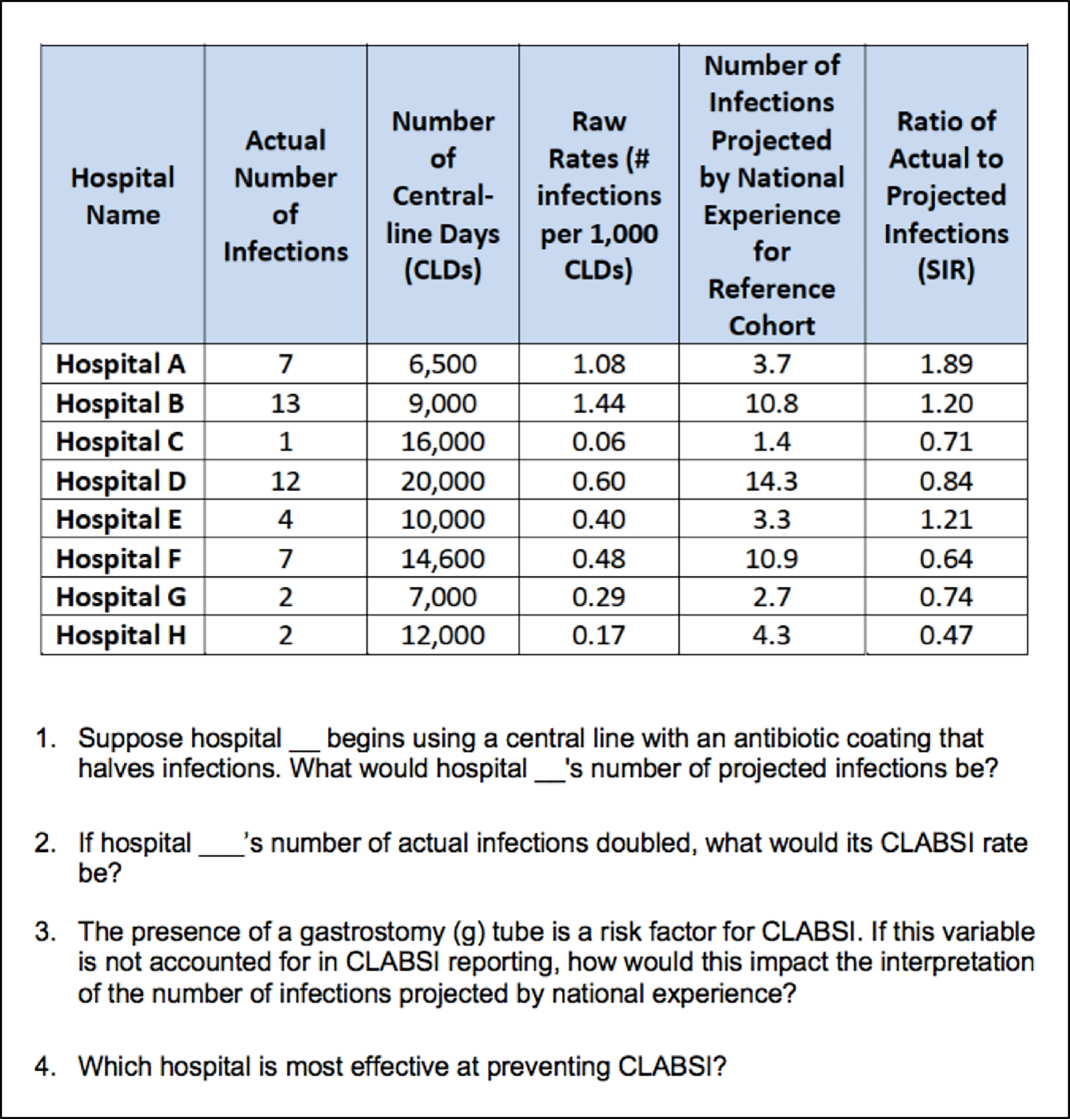
Final CLABSI comprehension scale based on factor analysis results.

During clinical interpretation, the solution was deemed an overall CLABSI “comprehension” scale for three reasons (Fig 2). First, the eigenvalue and Cronbach’s alpha suggested a strong and unidimensional construct. Second, all clinically relevant CLABSI data were represented within the items of the solution. Thus, the solution assessed the pertinent clinical concepts (e.g. raw rates, risk adjustment, overall performance) necessary for a CLABSI comprehension scale. Third, each item in the scale was free from semantic ambiguity based on cognitive interviewing with multiple informants. Therefore, the variance represented was felt to be true variation in comprehension rather than measurement error. We subsequently concluded that a general understanding of CLABSI data, or a CLABSI comprehension scale, was the construct represented in the final solution.

## Discussion

We present here the first psychometric evaluation of a scale that assesses clinician comprehension of quality metric data, with the specific application to central-line associated blood stream infections (CLABSI). In our sample of clinician responses, factor analysis resulted in a single factor solution that assessed overall CLABSI comprehension. Therefore, we recommend these 4 items could be used as a CLABSI comprehension scale. The scale is preliminary and would potentially benefit from further testing in other populations. However, this scale has the following virtues: internal consistency, assessment of clinically relevant concepts related to CLABSI comprehension, and parsimony that will assist in response rates.[33, 34] This CLABSI comprehension scale is also relevant given the recent evidence suggesting poor CLABSI quality metric comprehension among clinicians.[17] Leveraging this tool, CLABSI comprehension could be evaluated as a modifiable factor in the efforts to make the data more effective in driving practice change.

While research on quality metric comprehension is early in its development, there is literature on clinician interpretation of other complex concepts.[35–37] Most of the literature on these alternative topics (e.g. post-test probability, screening test interpretation) points to variable clinician understanding. However, these studies rarely employed a previously developed, internally consistent scale. The result is an inability to reliably evaluate the specific mechanisms related to data comprehension.[38, 39] Our research is unique in that it applies psychometric methodology while assessing comprehension of a complex clinical concept. The result is greater reliability in ensuring the measured variation is related to comprehension, not extraneous confounders.

Our scale and methodology used for derivation have important health policy implications. With the growing focus on pay for performance, there has been additional emphasis on rigorous collection and reporting of complex quality metrics.[40, 41] Nevertheless, these metrics as feedback agents are not reliably efficacious despite an estimated cost in the tens of billions of dollars.[5] Deficient comprehension of risk adjusted quality metric data is a plausible reason for this ineffectiveness. By providing a preliminary framework to reliably assess comprehension of quality metrics, this scale and its methodology could lead to more effective motivation of clinician behavior.

There are limitations to this study. First, the sample size for the survey is small in the context of psychometric methodology. However, our subject to item ratio was 10:1, which satisfies recommended standards by Costello as a marker of adequate sampling.[27] The sample size provided a dataset that met the screening criteria for factor analysis. Additionally, the sample was sufficient to provide a succinct and internally consistent scale on CLABSI comprehension. Second, this was a convenience sample of frontline clinicians recruited from Twitter. Thus, it is unclear how representative this sample is with respect to other clinician samples. It should be noted that these participants were Twitter-followers of two health services researchers (TJI, VC) who are actively engaged in scholarly research. Nevertheless, while the data provided the necessary variation for factor analysis, other samples (e.g., infection preventionists) might be relevant for scale refinement and additional research. Third, the scale is limited to CLABSI comprehension, and the methodology would need to be translated to other metrics for alternative assessments.

The results of this study have important implications for further research. CLABSI comprehension scale refinement should be performed in alternative populations for scale optimization. CLABSI comprehension assessments and scale deployment need to be carried out in policy-relevant populations (e.g., infection control practitioners, ICU directors). It is also prudent to evaluate whether CLABSI metric comprehension is linked to CLABSI outcomes. The mechanistic association between comprehension and behavioral response, while tenable, cannot be assumed given the complexity of behavior response.[42] However, research utilizing this scale could help elucidate what drives behavioral response, which is impossible without the reliable measurement of key constructs. Additionally, translating this methodology to other quality metrics is essential as policy experts seek to better leverage the information as practice change agents. Finally, the impact of alternate data presentations on quality metric comprehension and performance is worthy of further testing; indeed, varying infographic presentations have been shown to impact other forms of communication.[43, 44]

Data as feedback agents are ubiquitous in modern society as they inform and elicit behavioral responses. Inherent in this process is data comprehension, and reliable assessments of comprehension require scales.[38, 39] While these have been developed in other areas of research,[45, 46] this is not the case with quality metrics. Our paper provides a preliminary framework on how to assess clinician comprehension of metric data in the specific area of CLABSI, which may eventually be generalized to other areas. The methodology seeks to assist quality improvement initiatives in more effectively deploying these data as motivators of clinician behavior. The goal is a greater understanding of how quality metric feedback translates into practice change.

## Supporting information

S1 Fig Stata code. (PNG)

S1 Table Deidentified data set. (XLS)
