# Supplementary figures and images for: A comprehension scale for central-line associated bloodstream infection: Results of a preliminary survey and factor analysis
Source: PLoS One. 2018 Sep 13;13(9):e0203431. doi: 10.1371/journal.pone.0203431 (PMC6136729; doi:10.1371/journal.pone.0203431)

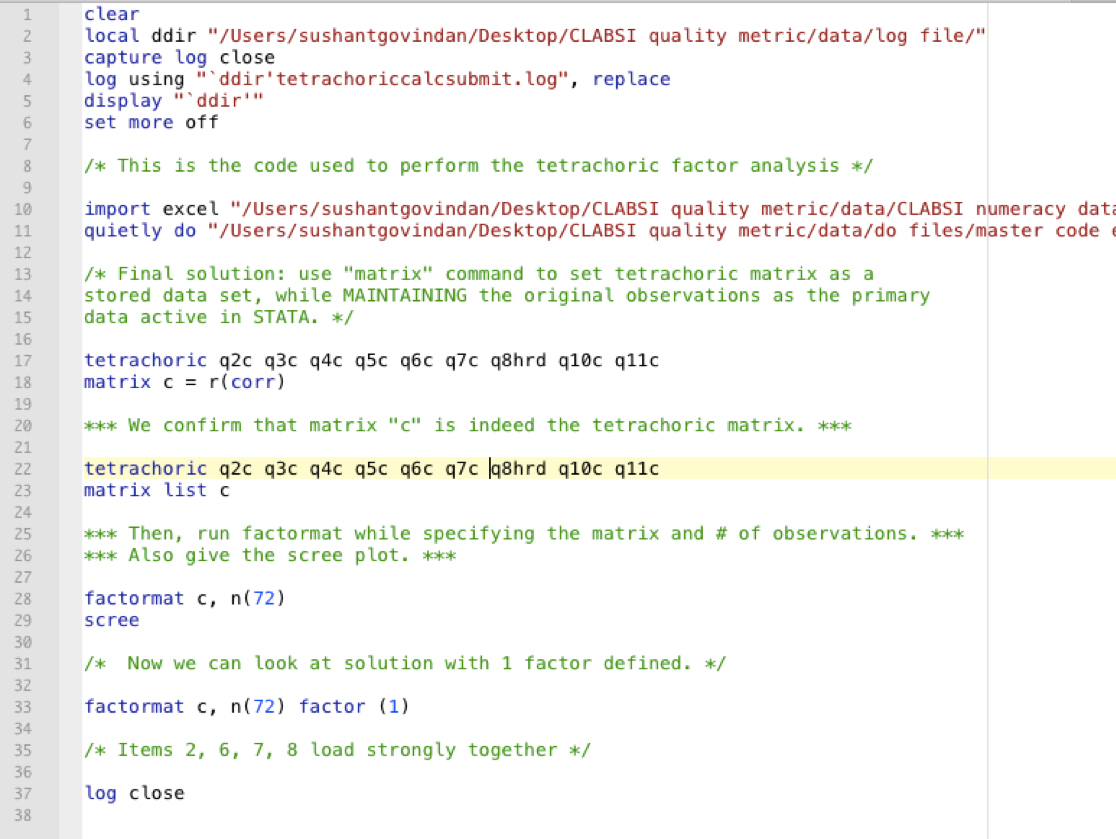

Supplement: S1 Fig — (PNG) [file pone.0203431.s001.png]
